# Help-seeking behaviours for psychological distress amongst Chinese patients

**DOI:** 10.1371/journal.pone.0185831

**Published:** 2017-10-02

**Authors:** Kai Sing Sun, Tai Pong Lam, Kwok Fai Lam, Leon Piterman, Tak Lam Lo, David Vai Kiong Chao, Edmund Wing Wo Lam

**Affiliations:** 1 Department of Family Medicine and Primary Care, The University of Hong Kong, Hong Kong, China; 2 Department of Statistics and Actuarial Science, The University of Hong Kong, Hong Kong, China; 3 Pro Vice-Chancellor and Professor of General Practice, Monash University, Melbourne, Australia; 4 Kwai Chung Hospital, Hong Kong, China; 5 Department of Family Medicine and Primary Health Care, United Christian Hospital and Tseung Kwan O Hospital, Hong Kong, China; The Chinese University of Hong Kong, HONG KONG

## Abstract

**Background:**

The stepped care model for psychological distress has been promoted in recent years, leading to the enhancing roles of primary care professionals and alternative sources of help. However, most of the research findings come from Western countries. This study investigates help-seeking behaviours of Chinese patients among different types of professional and alternative sources for psychological distress in Hong Kong.

**Methods:**

A questionnaire survey was conducted with 1626 adult primary care attenders from 13 private and 6 public clinics, 650 (40.0%) reported that they had ever experienced psychological distress. Their help-seeking behaviours, demographic background and current distress level (measured by GHQ-12) were analysed.

**Results:**

Among the respondents with experience of psychological distress, 48.2% had sought help from professional and/or alternative sources for their distress [10.2% from professionals only, 12.6% from alternative sources only, and 25.4% from both]. Those who had sought help from professionals only were more likely to be less educated and with lower income. In contrast, those using alternative sources only were more likely to be younger, better educated, and have higher income. Allowing multiple responses, psychiatrists (22.3%) was reported to be the most popular professional source, followed by primary care physicians (17.5%), clinical psychologists (12.8%) and social workers/counsellors (12.0%). Family members/friends (28.6%) was the top alternative source, followed by exercise/sports (21.8%), religious/spiritual support (16.9%) and self-help websites/books/pamphlets (8.9%).

**Conclusion:**

While psychiatrists remain the most popular professional source of help to the Chinese patients in Hong Kong, primary care professionals and alternative sources also play significant roles. Distressed patients who are younger, better educated and have higher income are more likely to use alternative sources only. The outcomes need further research.

## Introduction

In recent years, the World Health Organization has promoted primary care for mental health in response to the high prevalence of depression and anxiety disorders [1]. The stepped care model has been implemented in the UK, the Netherlands, Australia and New Zealand [2, 3]. It emphasises the least intensive intervention required at the beginning and the patient could step up or down the pathway according to treatment outcome [4]. This leads to enhancing roles of primary care physicians (PCPs), social workers, counsellors in managing psychological distress. The Australian National Survey of Mental Health and Wellbeing reported that 24.7% of mental health patients were treated by PCPs, 13.2% by psychologists, 7.9% by psychiatrists, 7.7% by other mental health professionals, and 6.6% by other health professionals (including specialist doctors, other professionals providing general services, and complementary/alternative therapists). Some patients received help from more than one source [5]. Similar pattern of service use was observed in a US national survey [6]. On the other hand, there is a rise in the role of alternative sources of help such as exercise, spiritual support, self-help websites and books [7–9]. Research studies demonstrated the positive effects of alternative sources [10–12] but only few help-seeking preference surveys included them in the comparison with professional sources [7, 9].

Despite the well-developed stepped care model in the UK and Australia, integration of mental health care into primary care is at the stage of infancy in many other countries including China. A recent mental morbidity survey in Hong Kong found that the highest proportion of people with common mental disorders consulted psychiatrists (13.8%), followed by social workers/counsellors (9.3%), PCPs (5.9%) and psychologists (3.9%) [13]. Similar to the US and most Asian countries [14], the health care system in Hong Kong has a mixed mode of public-private financing [15]. The general public can consult any private specialists including psychiatrists without a PCP referral. This may contribute to the higher rate of help-seeking from psychiatrists than PCPs. Regarding alternative sources, another local help-seeking survey based on a depression vignette reported that 78.7%, 75.6% and 69.4% of general public respondents would seek help from friends, family and religious practitioners respectively. Besides, 93.1% thought that exercise would be helpful [16]. Nonetheless, their actual help-seeking behaviours were not investigated.

This study was part of a large project investigating the help-seeking attitudes and behaviours of the Chinese for psychological distress in Hong Kong. Psychological distress refers to an emotional state characterised with anxiety and/or depressive symptoms [17]. The findings regarding their barriers to seeking help have been published elsewhere [18]. The current paper aims to investigate their help-seeking behaviours among different types of professional and alternative sources, and the association with their demographic characteristics. As the Chinese, either living within or outside China, represent one fifth of the world’s population, many doctors and other health professionals around the world have the chance to look after Chinese patients. For instance, in the US, there are over 3.3 million Chinese Americans who are the largest Asian group in the country [19]. There are similar situations in Canada, Australia and other countries with multi-cultural diversity. Our findings will be useful for policy makers and different service providers to enhance mental health care for the Chinese patients.

## Methods

### Sample

A cross-sectional survey was conducted among primary care attenders between October 2013 and August 2014. The target population was Chinese patients aged 18 or over attending primary care services. One objective of the survey was to determine the proportion of primary care attenders with psychological distress who would seek help. Without any information about this proportion p, we made use of the most conservative choice with p = 0.5. To ensure the estimation error would be at most 0.05 with 95% confidence, a sample size of 385 was required [20]. As reported by a WHO international study [21] that over 25% of primary care consultations had a significant psychological component, we needed to have at least n = 385/0.25 = 1540 primary care attenders to be recruited. A total of 1626 subjects successfully completed the questionnaires, with about half recruited from private primary care and the other half from public primary care settings for comparison purpose. The respondents were recruited from various districts over the Hong Kong territory to cover different demographics of the population. One out of three attenders at the clinic waiting area was invited by research assistants to complete the questionnaire. Written consent was obtained from the survey participants. Primary care attenders who had significant hearing difficulty, intellectual disability or were not able to communicate in Chinese were excluded. Most participants completed the questionnaire by themselves. For some elderly participants who had difficulties in reading, the research assistants helped to administer the questionnaire. To encourage responses, HK$20 (US$2.6) was offered to each respondent as incentive. Ethics approvals were obtained from the Institutional Review Board of The University of Hong Kong / Hospital Authority Hong Kong West Cluster (UW 09–326) and the Research Ethics Committee of Kowloon Central Cluster / Kowloon East Cluster (KC/KE-13-0091).

### Questionnaire

A help-seeking questionnaire containing questions on help-seeking behaviours for psychological distress was developed based on the themes identified from previous focus groups, individual interviews, as well as literature review [7]. The questionnaire was pilot-tested for its face- and content-validity with 8 laymen. All subjects rated most of the items as comprehensible and relevant. Minor modifications were made based on feedbacks, and the final questionnaire was further tested with 28 patients. A Cronbach's alpha coefficient of 0.725 for the question items was achieved based on the pilot sample, which was considered to be sufficient to demonstrate internal consistency. In the questionnaire, psychological distress was defined as an emotional state characterised with anxiety and/or depressive symptoms. Lists of professional and alternative sources were provided to the respondents to select the sources which they had used. The professional sources included psychiatrists, clinical psychologists, PCPs, traditional Chinese medicine (TCM) practitioners, social workers/counsellors, psychiatric nurses and primary care nurses. The alternative sources included family members/friends, religious/spiritual support, self-help websites/books /pamphlets, support groups, and exercise/sports. Demographic questions were also asked (please see S1 File for the question items reported in this article). In addition to the help-seeking questionnaire, GHQ-12 screening questionnaire was used to identify primary care attenders with different degrees of psychological distress recently, the responses would be correlated with their corresponding responses from the help-seeking questionnaire. GHQ-12 is a widely used and well-validated screening instrument for psychological distress and its Chinese version has also been validated [22]. The GHQ-12 consisted of a checklist of statements on psychological well-being asking respondents to compare their recent experience to their usual state with a score 0 if the condition is no worse than usual and a score of 1 if the condition is worse than usual. The total score ranged from 0 to 12, with higher scores representing more symptoms of psychological distress [23, 24]. A total score of 4 or higher indicated high risk of distress [23, 25].

### Statistical analysis

The quantitative data were analysed using JMP (Release 10.0.0). We used frequencies and percentages to summarize the responses of the question items. Pearson Chi-squared test and Kruskal-Wallis test were carried out to determine the differences in help seeking behaviours of participants for nominal demographic variables, namely gender and health care setting, and ordinal demographic variables, namely age, education, income and GHQ score, respectively. A p-value < 0.05 was considered statistically significant.

## Results

### Participants recruited

Excluding 22 incomplete interviews (major sections unanswered), there was a total of 1626 successfully completed questionnaires. The response rate of eligible subjects was 72.3%. Out of the 1626 respondents, 847 were recruited from 13 private clinics (52.1%) and 779 (47.9%) from 6 public clinics. Their age and household income distributions were similar to the Hong Kong population as reported in the 2011 Census. Details of their demographic characteristics had been reported elsewhere [18].

### Overall help-seeking pattern

Out of the 1626 respondents, 650 (40.0%) reported that they had ever experienced psychological distress. Among these 650 respondents, 313 (48.2%) had sought help from professional and/or alternative sources for their psychological distress. The background characteristics of these 650 respondents with and without help-seeking experiences are compared in Table 1. No significant difference between the two groups was shown.

**Table 1:** Comparison of background characteristics between respondents with and without help-seeking experience for psychological distress.

	Had sought help (N = 313)		Had NOT sought help (N = 337)		Pearsonχ^2^ test / Wilcoxon rank-sum test^a^
	n	(%)	n	(%)	P-value
**Gender**					
male	77	(26.1)	98	(30.7)	0.205
female	218	(73.9)	221	(69.3)	
**Age**					
18–29	51	(16.5)	83	(24.9)	0.110
30–39	91	(29.4)	76	(22.8)	
40–49	63	(20.4)	67	(20.1)	
50–59	63	(20.4)	80	(24.0)	
60 or over	41	(13.3)	27	(8.1)	
**Education**					
primary	28	(9.1)	29	(8.7)	0.435
secondary	146	(47.4)	171	(51.5)	
tertiary	134	(43.5)	132	(39.8)	
**Income**					
low	118	(40.7)	123	(38.1)	0.759
middle	111	(38.3)	136	(42.1)	
high	61	(21.0)	64	(19.8)	
**GHQ score**					
score 0–1	101	(32.4)	99	(29.4)	0.299
score 2–3	67	(21.5)	101	(30.0)	
score 4–6	79	(25.3)	93	(27.6)	
score 7 or over	65	(20.8)	44	(13.1)	
**Health care setting**					
private	168	(53.7)	159	(47.2)	0.098
public	145	(46.3)	178	(52.8)	

^a^Pearsonχ^2^ test for the variables gender and health care setting, and Wilcoxon rank-sum test for the other variables

Some data in the categories were missing due to respondents’ refusal to answer or invalid response

Among the 650 respondents with distress experience, 66 (10.2%) had sought help from professionals only, 82 (12.6%) from alternative sources only, and 165 (25.4%) from both sources. Their background characteristics are compared in Table 2. Significant differences among the three groups for the variables age (p<0.001), education (p<0.001) and income (p<0.001) were shown by Kruskal-Wallis test. Those who had sought help from alternative sources only were more likely to be younger, better educated, and have higher income. In contrast, those who had sought help from professionals only were more likely to be less educated and with lower income.

**Table 2:** Comparison of background characteristics among respondents who had sought help from professionals only, alternative sources only, or both.

	Professionals only (N = 66)		Alternative sources only (N = 82)		Both (N = 165)		Pearson χ^2^ test / Kruskal-Wallis test^a^
	n	(%)	n	(%)	n	(%)	P-value
**Gender**							
male	16	(25.0)	19	(24.4)	42	(27.5)	0.857
female	48	(75.0)	59	(75.6)	111	(72.5)	
**Age**							
18–29	9	(13.8)	24	(29.3)	18	(11.1)	<0.001**
30–39	16	(24.6)	23	(28.0)	52	(32.1)	
40–49	12	(18.5)	21	(25.6)	30	(18.5)	
50–59	17	(26.2)	8	(9.8)	38	(23.5)	
60 or over	11	(16.9)	6	(7.3)	24	(14.8)	
**Education**							
primary	7	(10.8)	5	(6.1)	16	(9.9)	<0.001**
secondary	41	(63.1)	25	(30.5)	80	(49.7)	
tertiary	17	(26.2)	52	(63.4)	65	(40.4)	
**Income**							
low	37	(62.7)	21	(25.9)	60	(40.0)	<0.001**
middle	16	(27.1)	39	(48.1)	56	(37.3)	
high	6	(10.2)	21	(25.9)	34	(22.7)	
**GHQ score**							
score 0–1	22	(33.3)	23	(28.0)	56	(34.1)	0.726
score 2–3	11	(16.7)	21	(25.6)	35	(21.3)	
score 4–6	16	(24.2)	22	(26.8)	41	(25.0)	
score 7 or over	17	(25.8)	16	(19.5)	32	(19.5)	
**Health care setting**							
private	35	(53.0)	43	(51.8)	93	(54.1)	0.943
public	31	(47.0)	40	(48.2)	79	(45.9)	

**p<0.001

^a^Pearsonχ^2^ test for the variables gender and health care setting, and Kruskal-Wallis test for the other variables

Some data in the categories were missing due to respondents’ refusal to answer or invalid response

### Help-seeking pattern for specific types of professional and alternative sources

We analysed their help-seeking pattern for specific types of professional and alternative sources. They were allowed to select multiple sources used. The combined and separated help-seeking patterns of respondents attending private and public primary care clinics are shown in Table 3. Considering the combined help-seeking pattern of the two groups, psychiatrists (22.3%) was the most popular professional source, followed by PCPs (17.5%), clinical psychologists (12.8%) and social workers/counsellors (12.0%). TCM practitioners (5.1%), psychiatric nurses (4.3%) and primary care nurses (2.9%) were less popular sources. Family members/friends (28.6%) was the top alternative source, followed by exercise/sports (21.8%), religious/spiritual support (16.9%) and self-help websites/books/pamphlets (8.9%). A small proportion of respondents used support groups (4.3%).

**Table 3:** Comparison of help-seeking pattern for specific sources between respondents attending private and public primary care clinics.

	Private primary care attender (N = 327)		Public primary care attender (N = 323)		Pearson χ^2^ test	Total (N = 650)	
	n	(%)	n	(%)	P-value	n	(%)
***Professionals***							
Psychiatrists	68	(20.8)	77	(23.8)	0.351	145	(22.3)
Clinical psychologists	41	(12.5)	42	(13.0)	0.859	83	(12.8)
PCPs	69	(21.1)	45	(13.9)	0.016*	114	(17.5)
TCM practitioners	11	(3.4)	22	(6.8)	0.045*	33	(5.1)
Social workers/ counsellors	32	(9.8)	46	(14.2)	0.081	78	(12.0)
Psychiatric nurses	6	(1.8)	22	(6.8)	0.002*	28	(4.3)
Primary care nurses	5	(1.5)	14	(4.3)	0.034	19	(2.9)
***Alternative sources***							
Family members/ friends	102	(31.2)	84	(26.0)	0.143	186	(28.6)
Religious/ spiritual support	50	(15.3)	60	(18.6)	0.264	110	(16.9)
Self-help websites, books or pamphlets	25	(7.6)	33	(10.2)	0.250	58	(8.9)
Support groups	8	(2.4)	20	(6.2)	0.019*	28	(4.3)
Exercise and sports	73	(22.3)	69	(21.4)	0.767	142	(21.8)

Multiple responses allowed

*p<0.05

**Fig 1:**
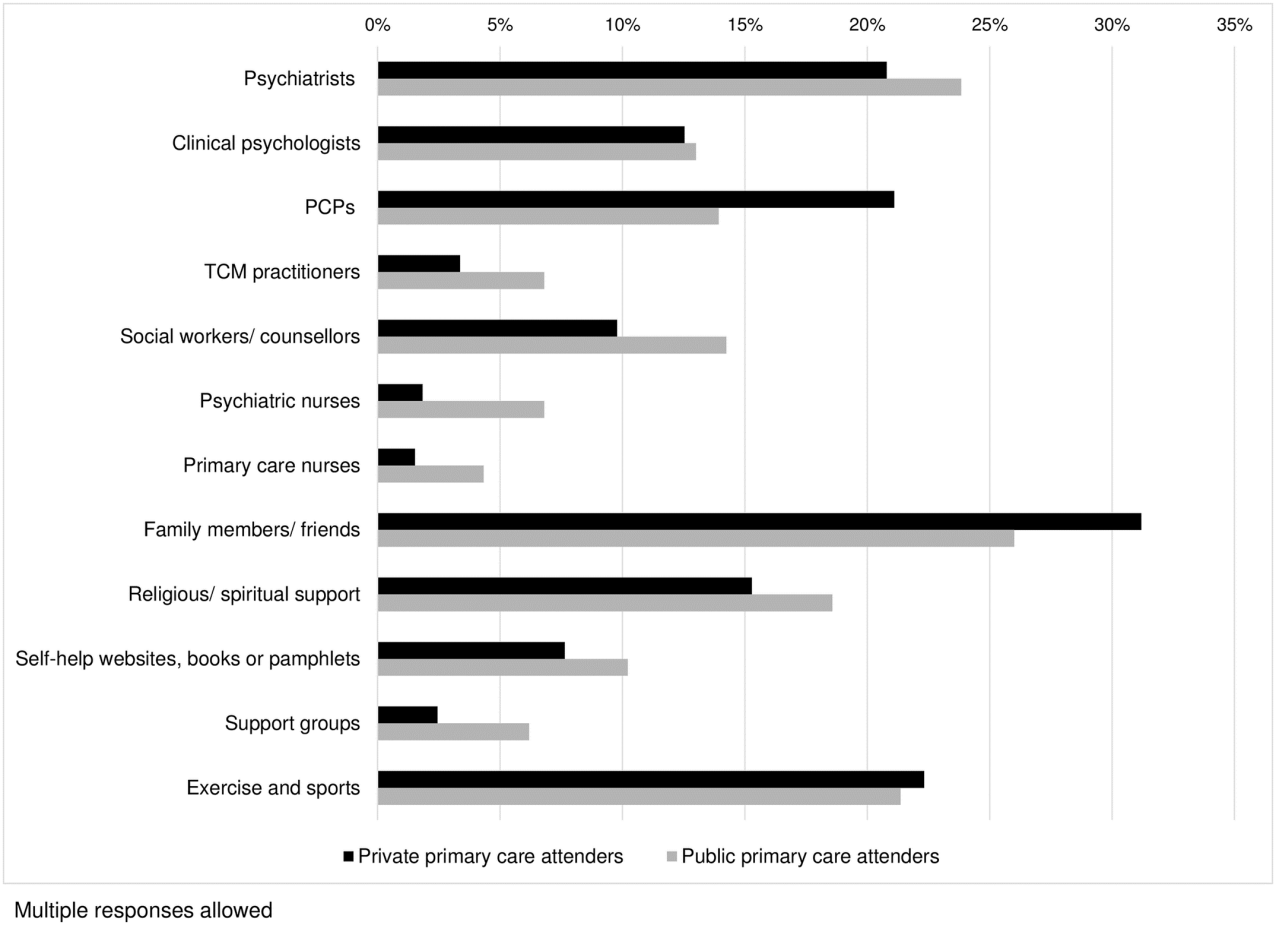
Comparison of the help-seeking pattern for specific sources between respondents attending private (N = 327) and public (N = 323) primary care clinics.

Comparing the separated help-seeking patterns between the private and public primary care attenders, significant differences in responses were shown by Pearson Chi-squared test for the sources PCPs (p = 0.016), TCM practitioners (p = 0.045), psychiatric nurses (p = 0.002), primary care nurses (p = 0.034) and support groups (p = 0.019). The public primary care attenders were less likely to seek help from PCPs (private: 21.1%; public: 13.9%), but more likely to seek help from TCM practitioners (private: 3.4%; public: 6.8%), psychiatric nurses (private: 1.8%; public: 6.8%), primary care nurses (private: 1.5%; public: 4.3%) and support groups (private: 2.4%; public: 6.2%). Fig 1 presents a bar chart contrasting the help-seeking patterns between private and public primary care attenders.

## Discussion

The effect of demographic factors was shown in help-seeking behaviours. Patients who had sought help from professionals only were more likely to be less educated and with lower income. Those who had sought help from alternative sources only were more likely to be younger, better educated, and have higher income. This finding indicates that professional and alternative sources can complement each other to cover a wide-spectrum of patients with different demographic characteristics. Health literacy may influence their help-seeking behaviours. The better educated are likely to search on the internet for alternative therapies [26] and self-manage in contrast to the less educated who rely on conventional sources of help. In fact, the younger adults were shown to be less likely to seek professional help in our Chinese sample as well as in Western samples [5, 27]. In addition to formal primary care services, alternative sources may be considered as the starting point of stepped care [4]. This may increase access to mental health care and reduce stigma of help-seeking. Mental health literacy and self-efficacy can be promoted by public education campaigns such as Beyondblue in Australia [28] and Time to Change in the UK [29]. There is also a local three-year drive, Joyful@HK Campaign, launched in January 2016 [30]. Its effects are yet to be evaluated.

Alternative sources have a significant role to Chinese patients who have barriers to seeking professional care such as worries of being prescribed psychiatric drugs by doctors, stigma, accessibility of services and treatment cost [18]. Nonetheless, transition from alternative to professional sources should be ensured if stronger intervention is needed. The distress level of our survey respondents before treatment could not be reflected by their current GHQ scores. We found that family/friends was the most popular choice of alternative sources, followed by exercise and religious support. It is known from the literature that emotional support from family and friends was associated with lower psychological distress [31]. But it is questionable if they could handle cases of persistent negative thinking patterns or avoidance behaviours without a systematic management plan. Similar concerns are held for using exercise or religious support alone. Studies showed both positive and negative effects of religious coping [32, 33]. Praying might sometimes be used as a safety behaviour to neutralize anxiety. In Hong Kong, we had a series of school and university students committing suicide in the recent past [34]. Further research is needed to investigate whether the youth are able to self-manage. Besides, the quality of self-help information such as credibility of sources, supporting evidence, and adaptability for a Chinese context are important factors. In fact, the websites can also be the connection points to professional sources such as PCPs, psychologists, social workers, psychiatrists, support hotlines and community mental health centres, like what is done by Headspace in Australia [35].

Our findings on the usage pattern of specific types of professional and alternative sources can be compared with that of Western surveys. Unlike the findings in Australia and the US [5, 6], we had more patients seeing psychiatrists than PCPs and psychologists. For alternative sources, we found a similar pattern that the largest proportion of respondents sought help from family/friends, followed by exercise. However, our respondents showed a stronger preference for religious/spiritual support than the Western samples in Australia and European countries [7, 9, 36]. Within the health care system of Hong Kong, we found differences in the types of sources used by the private and public primary care attenders. The public primary care attenders were less likely to seek help from PCPs. Our previous findings indicated that patients perceived stronger barriers in help seeking for psychological distress in public primary care clinics. One highlighted barrier was about not having a regular PCP [18]. On the other hand, the current study results showed that the public clinic attenders had higher usage of the sources of TCM practitioners, psychiatric nurses, primary care nurses and support groups for mental health care. This indicates the stronger resource support and interprofessional collaboration in the public setting. Besides, mental health training programmes for TCM practitioners in the public setting have been conducted since 2010 [37]. The policy makers should attend to the strengths and limitations of the public setting in mental health care.

This study had several limitations. Firstly, the survey findings were based on self-reported data from the respondents. However, the potential recall bias should be small as the questions were about their personal experiences. Secondly, the severity of their distress before seeking help could not be reflected by their current GHQ scores. Thirdly, the interpretations of the findings were mainly based on demographic factors and GHQ scores. Other factors such as quality of services, accessibility, stigma, acceptability of drug treatment, and public education were discussed in our previous paper [18]. Lastly, the survey respondents were primary care attenders. The results might not be generalizable to the general public in Hong Kong.

## Conclusions

While psychiatrists remain the most popular professional source of help for psychological distress to the Chinese in Hong Kong, primary care professionals and alternative sources play significant roles. Health literacy may influence their help-seeking behaviours. Distressed patients who are younger, better educated, and have higher income are more likely to use alternative sources of help, while the less educated are more likely to rely on professional sources. Thus, in addition to formal primary care services, alternative sources may be seen as the starting point of stepped care, especially for the youth and the educated. However, outcome of self management in a Chinese context needs further research. Transition from alternative to professional sources should be facilitated if stronger intervention is needed. Regarding professional sources, the public primary care attenders use a wider range of mental health services but are less likely to seek help from PCPs compared with the private attenders.

## Supporting information

S1 File Help-seeking behaviour questions. (DOCX)
