# Supplementary material for: Help-seeking behaviours for psychological distress amongst Chinese patients
Source: PLoS One. 2017 Oct 2;12(10):e0185831. doi: 10.1371/journal.pone.0185831 (PMC5624640; doi:10.1371/journal.pone.0185831)
Supplement: S1 File — (DOCX) [file pone.0185831.s001.docx]

問卷編號 Questionnaire No.:

**這份問卷會詢問一些有關你的求診模式及你對有情緒困擾的人士尋求協助的意見。**

**註：情緒困擾是指一種帶有焦慮或抑鬱徵狀的情緒狀態。**

**This questionnaire will ask you a range of questions about your consultation pattern and views on the psychological distressed to seek help. Psychological distress refers to an emotional state characterized with anxiety and/or depressive symptoms.**

| 你曾經遇過情緒困擾嗎？ (如感到焦慮、抑鬱等)  Have you ever experienced psychological distress (e.g. anxiety, depressive symptoms)?  □有 Yes □沒有 No [**跳過以下部份** Skip the following section] |
| --- |

**你有否因為情緒困擾而曾經求助下列的人士或方式？(請選擇答案A或B)**

**Have you ever used the sources or ways listed below to help for psychological distress? (Please choose either A or B)**

| □A. **有試過。** **Yes.**  **請在下列選取你曾試過的方法。**  **Please select from the listed sources below.** | | |
| --- | --- | --- |
|  |  | **求助方式**  **Sources to help** |
| 1. | □私家的Private  □政府 Public | 精神科醫生Psychiatrists |
| 2. | □私家的Private  □政府 Public | 臨床心理學家 Clinical psychologists |
| 3. | □私家的 | 普通科醫生/家庭醫生 General practitioners/ family doctors |
| 4. | □私家的 | 中醫 TCM practitioners |
| 5. | □私家的 | 社工或輔導員 Social workers or counselors |
| 6. | □精神科 Psychiatric  □普通科 Primary Care | 護士 Nurses |
| 7. | □私家的 | 家人或朋友 Family members or friends |
| 8. | □私家的 | 宗教/信仰 Religious or spiritual support |
| 9. | □私家的 | 自助小冊子、書籍或網站 Self-help websites, books or pamphlets |
| 10. | □私家的 | 互助小組 Support groups |
| 11. | □私家的 | 做運動 Exercise and sports |

| □B. **沒有試過。 No.** |
| --- |

**為了研究用途，我們希望你能提供一些個人資料。請放心，你提供的資料將會絕對保密。**

**Please tell us more about yourself in order to facilitate our analysis. All information collected will be treated in strictest confidence.**

**性別 Gender:**  □男Male □女Female

**年齡組別 Which of the following age groups do you fall into?**

|  | □18-19 | □20-24 | □25-29 | □30-34 | □35-39 |
| --- | --- | --- | --- | --- | --- |
|  | □40-44 | □45-49 | □50-54 | □55-59 | □60-64 |
|  | □65-69 | □70-74 | □75或以上/ or above | | |

**教育程度 What is the highest level of education you have achieved?**

|  | □無/幼稚園  No schooling/kindergarten | □小學程度  Primary |  |
| --- | --- | --- | --- |
|  | □中學程度(中一至中三)  (F.1-F.3 Lower secondary) | □中學程度(中四至中五)  (F.4-F.5 Upper secondary) | □預科  Matriculation |
|  | □專上教育: 非學位課程  Tertiary:non-degree | □專上教育: 學位課程  Tertiary: Degree |  |

**每月家庭總收入組別($) Approximately, how much is your monthly household income ($) including all the income?**

|  | □2000以下/ or below | □2000-3999 | □4000-5999 | □6000-7999 |
| --- | --- | --- | --- | --- |
|  | □8000-9999 | □10000-14999 | □15000-19999 | □20000-24999 |
|  | □25000-29999 | □30000-39999 | □40000-59999 | □60000或以上/ or above |
